# Environmental Antidepressants Disrupt Metabolic Pathways in *Spirostomum ambiguum* and *Daphnia magna*: Insights from LC-MS-Based Metabolomics

**DOI:** 10.3390/molecules30142952

**Published:** 2025-07-13

**Authors:** Artur Jędreas, Sylwia Michorowska, Agata Drobniewska, Joanna Giebułtowicz

**Affiliations:** 1 Department of Drug Chemistry, Pharmaceutical and Biomedical Analysis, Medical University of Warsaw, 02-091 Warszawa, Poland; arturjedreas@outlook.com; 2 Department of Toxicology and Food Science, Medical University of Warsaw, 02-091 Warszawa, Poland; agata.drobniewska@wum.edu.pl

**Keywords:** environmental contamination, environmental toxicology, untargeted metabolomics, mass spectrometry, pharmaceuticals

## Abstract

Pharmaceuticals such as fluoxetine, paroxetine, sertraline, and mianserin occur in aquatic environments at low yet persistent concentrations due to their incomplete removal in wastewater treatment plants. Although frequently detected, these neuroactive compounds remain underrepresented in ecotoxicological assessments. Given their pharmacodynamic potency, environmentally relevant concentrations may induce sublethal effects in non-target organisms. In this study, we applied untargeted LC-MS-based metabolomics to investigate the sublethal effects of four widely used antidepressants—paroxetine, sertraline, fluoxetine (SSRIs), and mianserin (TeCA)—on two ecologically relevant freshwater invertebrates: *S. ambiguum* and *D. magna*. Organisms were individually exposed to each compound for 48 h at a concentration of 100 µg/L and 25 µg/L, respectively. Untargeted metabolomics captured the sublethal biochemical effects of these antidepressants, revealing both shared disruptions—e.g., in glycerophospholipid metabolism and cysteine and methionine metabolism—and species-specific responses. More pronounced pathway changes observed in *D. magna* suggest interspecies differences in metabolic capacity or xenobiotic processing mechanisms between taxa. Among the four antidepressants tested, sertraline in *D. magna* and fluoxetine in *S. ambiguum* exerted the most extensive metabolomic perturbations, as evidenced by the highest number and pathway impact scores. In *D. magna*, fluoxetine and mianserin produced similar metabolic profiles, largely overlapping with those of sertraline, whereas paroxetine affected only a single pathway, indicating minimal impact. In *S. ambiguum*, paroxetine and mianserin elicited comparable responses, also overlapping with those of fluoxetine, while sertraline triggered the fewest changes. These results suggest both compound-specific effects and a conserved metabolic response pattern among the antidepressants used. They also underscore the considerable potential of metabolomics as a powerful and sensitive tool for ecotoxicological risk assessments, particularly when applied across multiple model organisms to capture interspecies variations. However, further research is essential to identify which specific pathway disruptions are most predictive of adverse effects on organismal health.

## 1. Introduction

Pharmaceuticals (PhACs) are increasingly detected in aquatic environments, where they may persist at low but sustained concentrations. These contaminants enter the environment through effluents, leachate, and improper drug disposal [1]. Due to their chemical stability, PhACs often persist in the environment at concentrations ranging from nanograms to micrograms per liter [2]. Particular concern has been raised over PhACs targeting the central nervous system, owing to their widespread usage and high pharmacodynamic potency, which enables them to exert biological activity at environmentally relevant concentrations. Many antidepressants have been detected in surface waters worldwide. For example, fluoxetine, one of the most frequently prescribed selective serotonin reuptake inhibitors (SSRIs), has been measured in freshwater ecosystems at concentrations ranging from <1 to 350 ng/L [3]. Other SSRIs, including paroxetine and sertraline, have also been commonly detected in aquatic environments. Paroxetine has been reported in surface waters (0.27–270 ng/L), rivers (2.7–110.98 ng/L), and groundwater (5.1–30.2 ng/L) across various monitoring studies [4,5,6,7,8,9,10], with concentrations as high as 3380 ng/L detected in wastewater treatment plant (WWTP) effluents from pharmaceutical manufacturing facilities [11]. This highlights the potential for localized environmental hotspots of contamination. Even higher levels, reaching up to 39.73 μg/L, have been reported in untreated municipal sewage [12]. Sertraline has been measured in freshwater samples globally at concentrations ranging from 0.77 to 120 ng/L [13,14,15]. In contrast, mianserin, an atypical tetracyclic antidepressant (TeCA), has been detected in surface waters at concentrations of up to 29 ng/L [8].

Many PhACs are present across ecosystems at concentrations below the thresholds typically associated with effects that can be detected by standard acute or chronic toxicity assays (e.g., mortality or reproductive impairment). However, this does not preclude their potential to induce subtle yet biologically relevant effects. Therefore, recent studies have increasingly focused on alternative endpoints such as metabolic rate, ionoregulation, oxidative stress, and metabolomic alterations, which can reveal subtle physiological disruptions caused by environmentally relevant concentrations of antidepressants [16]. Environmental metabolomics is increasingly recognized as a key approach for elucidating the sublethal mechanisms of action of PhAC contaminants in non-target aquatic organisms, offering greater sensitivity than traditional endpoints such as mortality, growth, or reproduction and enabling the identification of disruptions in pathways related to energy metabolism, neurotransmission, and homeostatic regulation [17,18]. Namely, carbamazepine, gabapentin, metformin, and propranolol have been shown to alter energy metabolism, amino acid biosynthesis, and oxidative stress responses in aquatic invertebrates such as *D. magna* and ostracods [19,20]. Endocrine-active compounds such as drospirenone, 17α-ethinylestradiol, and diclofenac have been associated with disruptions in amino acid, lipid, and energy metabolism in mussels, crucian carp, and freshwater snails [21,22,23].

Relatively few studies have investigated the impact of antidepressants [24,25,26], particularly regarding their impact on metabolic pathways in early trophic-level species such as protozoa and cladocerans. In an untargeted metabolomics analysis, *mytilus galloprovincialis* was exposed to citalopram (500 ng/L), both alone and in combination with polyethylene microplastics [27]. The study revealed significant modulation of specific metabolic pathways in the digestive gland tissue. Notably, the study demonstrated that PhAC exposure led to alterations in neurotransmitter metabolism (e.g., increased serotonin post-depuration), steroid and prostaglandin pathways (upregulation of PGD2/E2, hydroxypregnenolone), purine metabolism (modulated inosine monophosphate and uridine monophosphate), and partial overlap with TCA cycle disruptions. In a different targeted metabolomics study on *Carassius auratus* exposed to fluoxetine (100 ng/L), accumulation of fluoxetine and its metabolite norfluoxetine occurred in the brain and liver [28]. This led to decreased serotonin levels, reduced acetylcholinesterase activity, and downregulation of key metabolic enzymes (CYP1A, CYP3A, and GSTπ), indicating disruption of neuroendocrine signaling and detoxification capacity.

This study applied liquid chromatography coupled to mass spectrometry (LC-MS/MS) to investigate the metabolic responses of the protozoan *S. ambiguum* and the freshwater crustacean *D. manga* to selected antidepressants, i.e., selective serotonin reuptake inhibitors (paroxetine, sertraline, and fluoxetine) and a tetracyclic antidepressant (mianserin). Both organisms are ecologically relevant and commonly used in ecotoxicological studies. *S. ambiguum* inhabits aquatic environments and is noted for its tolerance to a range of pH levels [29], while *D. magna* is a model species for water quality assessments; its use is recommended by EMA [30]. Through untargeted metabolomic analysis, we aimed to identify the biochemical pathways affected by PhAC exposure, contributing to a mechanistic understanding of their impacts on aquatic invertebrates.

## 2. Results

Group-level differences were statistically evaluated using one way ANOVA with a false discovery rate (FDR) cutoff of 0.05, identifying 62 significant metabolites in *D. magna* and 113 in *S. ambiguum*.

### 2.1. Multivariate Analysis Reveals Exposure Specific Metabolic Shifts

**Figure 1:**
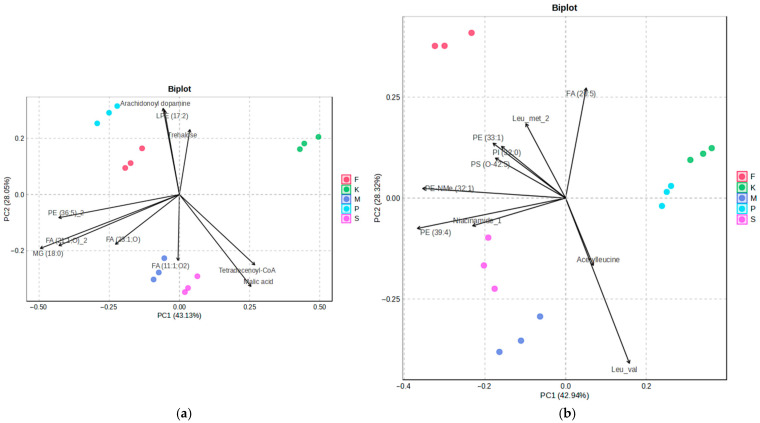
PCA biplots of metabolic profiles in (**a**) *S. ambiguum* and (**b**) *D. magna* exposed to antidepressants. LPE—lysophosphatidylethanolamine; PE—phosphatidylethanolamine; FA—free fatty acid; MG—monoacylglycerol; PS—phosphatidylserine. In lipid annotations, the number in parentheses refers to the total number of carbon atoms in the fatty acyl chains and the total number of double bonds.

PCA was used to explore global metabolic variation in response to antidepressant exposure in *D. magna* and *S. ambiguum*. In both species, the 2D scores plots showed clear separation between treatment groups. Biplots illustrating the main results of PCA are shown in Figure 1.

The PCA of the metabolic profiles of *S. ambiguum* and *D. magna* exposed to different antidepressants revealed clear separation between treatment groups along the first two principal components (PC1 and PC2), which together explained 71.18% and 71.26% of the total variance, respectively. Replicates clustered tightly within each treatment group, indicating consistent metabolic responses. In *S. ambiguum*, sertraline- and mianserin-treated samples showed the greatest separation from controls along PC2. These treatments were characterized by reduced levels of trehalose, LPE (17:2), and arachidonoyl dopamine and higher abundance of FA (11:1;O2). Along PC1, the most pronounced shifts relative to control samples were observed for paroxetine and fluoxetine. Paroxetine-treated samples exhibited the lowest abundances of tetradecenoyl-CoA and malic acid, while fluoxetine-treated samples also showed reduced levels of these metabolites, though to a lesser extent. Samples exposed to sertraline and mianserin clustered closely in the PCA scores plot, indicating similar metabolic responses to these antidepressants. This reflects biochemical similarity in how *S. ambiguum* processes or responds to these structurally different compounds.

In *D. magna*, the control group was distinct from all PhAC-treated groups, with fluoxetine exposure causing the largest shift. This shift was characterized by elevated levels of PI (31:0), PI (32:0), and PS (0–42:5) and the lowest level of Leu-val. Among the tested compounds, paroxetine-exposed *D. magna* showed the smallest PCA distance from controls, suggesting minimal metabolic disruption. In contrast, mianserin- and sertraline-treated samples elicited the most similar metabolic profiles, indicating that these PhACs may induce comparable toxic effects and share overlapping biochemical targets in *D. magna*. Notably, elevated levels of PE NMe (32:1) and PE (39:4) were consistently observed in organisms exposed to mianserin, sertraline, and fluoxetine.

The differences observed between the two test organisms suggested that the metabolic effects of these antidepressants are species-specific and reflect fundamental biological divergence between unicellular protozoa and multicellular cladocerans.

**Figure 2:**
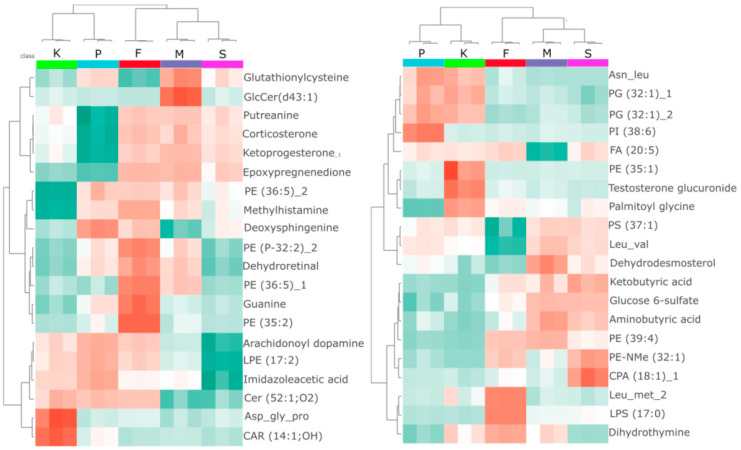
Heatmaps of 20 significantly altered metabolites, selected based on ANOVA, in *S. ambiguum* (**left**) and *D. magna* (**right**) following fluoxetine (F), mianserin (M), paroxetine (P), and sertraline (S) exposure. K-control group. Red indicates higher abundance compared to other exposure conditions, while green represents lower relative abundance. Complete datasets are presented in Figures S1 and S2.

The heatmaps presented in Figure 2 demonstrate clear clustering of organisms exposed to different PhACs. The results confirm the grouping of mianserin and sertraline. In *S. ambiguum*, exposure to any of the tested PhACs led to a decrease in the levels of the tripeptide Asp-gly-pro and CAR (14:1;OH). In contrast, in *D. magna*, exposure to PhACs resulted in a reduction of PE (35:1). Substance-specific effects were also observed. For example, *S. ambiguum* exposed to mianserin showed a significant increase in GlcCER (d43:1), while fluoxetine exposure led to elevated levels of guanine and PE (35:2). In *Daphnia*, sertraline increased CPA (18:1), paroxetine elevated PI (38:6), and fluoxetine induced an increase in compounds such as LPS (17:0).

### 2.2. Pathway-Level Metabolic Alterations in D. magna Following Antidepressant Exposure

**Figure 3:**
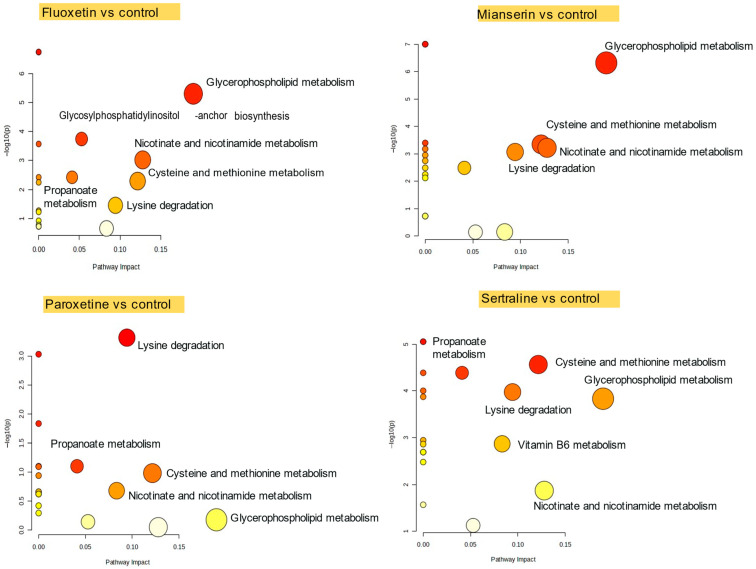
Significantly altered metabolic pathways in *D. magna* following fluoxetine, mianserin, paroxetine, and sertraline exposure. Circle color represents statistical significance, decreasing from red to yellow; circle size corresponds to pathway impact values.

Pathway analysis showed that exposure to fluoxetine, mianserin, paroxetine, and sertraline disrupted multiple metabolic pathways in *D. magna* (Figure 3). Glycerophospholipid metabolism was affected in animals exposed to sertraline, mianserin, and fluoxetine, indicating changes in cell membrane composition or lipid signaling [31]. Lysine degradation was consistently impacted across all treatments (borderline significance for fluoxetine, FDR = 0.058), suggesting alterations in amino acid catabolism and mitochondrial function. Nicotinate and nicotinamide metabolism, linked to NAD^+^ synthesis and redox processes, was perturbed by fluoxetine, mianserin, and sertraline (FDR < 0.05). Additional disruptions were observed in cysteine and methionine metabolism following PhAC exposure (with high impact (0.12), but no significance for paroxetine, FDR = 0.25). These results indicate that antidepressants interfere with core metabolic functions related to energy production, oxidative balance, and structural maintenance.

In *D. magna*, the 48-h EC_50_ values for immobility indicated that sertraline (1.15 mg/L) exhibited greater acute toxicity compared to fluoxetine (5.91 mg/L), paroxetine (6.24 mg/L), and mianserin (7.81 mg/L) [32]. The observed toxicity correlated with the abundance of fucose (r = −0.88, FDR = 0.037), Gly-met (r = 0.80, FDR = 0.037), PE-NMe (32:1) (r = −0.78, FDR = 0.037), and CPA (18:1) (r = −0.78, FDR = 0.037). PE-NMe (32:1) is a component of the glycerophospholipid pathway, which was affected by three out of four PhACs.

### 2.3. Pathway-Level Metabolic Alterations in S. ambiguum Following Antidepressant Exposure

**Figure 4:**
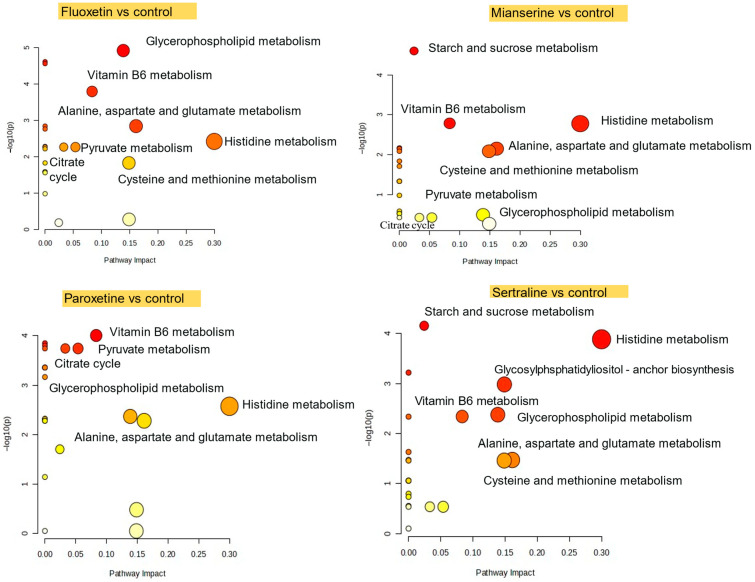
Significantly altered metabolic pathways in *S. ambiguum* following fluoxetine, mianserin, paroxetine, and sertraline exposure. Circle color represents statistical significance, decreasing from red to yellow; circle size corresponds to pathway impact values.

Pathway analysis revealed that fluoxetine, mianserin, paroxetine, and sertraline altered multiple metabolic pathways in *S. ambiguum* (Figure 4). Histidine metabolism as well as alanine, aspartate, and glutamate metabolism, were affected in all exposures (with high impact (0.16), but no significance for sertraline (FDR = 0.063) for the latter pathway), indicating disruptions in protein synthesis and oxidative defense, as well as in the histaminergic system and in energy homeostasis. Cysteine and methionine metabolism was significantly altered in the fluoxetine and mianserin groups, while the sertraline group showed borderline significance (FDR = 0.063). Glycerophospholipid metabolism was affected in animals exposed to sertraline, paroxetine, and fluoxetine, indicating changes in cell membrane composition or lipid signaling. These results show that antidepressant exposure affects core metabolic functions in *S. ambiguum*, particularly those related to membrane structure, oxidative balance, and energy pathways.

The 48-h EC_50_ (sublethal responses such as shortening, bending of the cell, immobilization) for various antidepressants for *S. ambiguum* were determined as follows: 0.35 mg/L for sertraline, 1.39 mg/L for fluoxetine, 1.10 mg/L for paroxetine, and 1.55 mg/L for mianserin [33]. In the current research, we observed strong and statistically significant positive correlation between the LC50 of the four PhACs and an abundance of histamine (r = 0.84, FDR = 0.011), PE (O-35:2) (r = 0.84, FDR = 0.011), glycerylphosphorylethanolamine (r = 0.76, FDR = 0.045), ketoprogesterone (r = 0.76, FDR = 0.045), and PE (P-32:2) (r = 0.75, FDR = 0.047). In contrast, a negative correlation was found for PA (42:9) (r = −0.94, FDR < 0.001), palmitoylethanolamide (r = −0.90, FDR = 0.0028), TG (45:2) (r = −0.88, FDR = 0.0033), PA (28:0) (r = −0.82, FDR = 0.018), and dimethylarginine (r = −0.78, FDR = 0.036). This group comprises compounds associated with the glycerophospholipid metabolic pathway, which has been shown to be disrupted by SSRI in *S. ambiguum*.

## 3. Discussion

One of the two organisms used in this study was *D. magna*, a planktonic microcrustacean which plays a vital ecological role in freshwater ecosystems, where it acts as a key trophic intermediary between primary producers and higher-level consumers [34]. It is an established model organism in regulatory toxicology [35], and it is widely used in aquatic toxicological studies to assess the effect of PhACs on non-target species [34]. Importantly, *Daphnia* shares a high number of genes with humans compared to other sequenced invertebrates [35]. Furthermore, its conserved neurotransmitter systems and associated gene pathways make it a robust model for investigating the effects of neuroactive environmental contaminants [36]. Three of the PhACs tested in our study are selective serotonin reuptake inhibitors (SSRIs), a class of antidepressants that alleviate depressive symptoms by increasing synaptic levels of serotonin (5-hydroxytryptamine; 5-TH). These compounds inhibit the serotonin transporter (SERT) at presynaptic nerve terminals, which is conserved across Metazoans, including *D. magna* [37]. Additionally, the presence of gene encoding enzymes involved in serotonin biosynthesis [37], endogenous serotonin [38], and serotonin-responsive neurons [39] in *Daphnia* further supports the relevance of this model organism for study of the effects of SSRIs in aquatic environments.

Fluoxetine has been shown to affect multiple biological endpoints in *D. magna*, including growth, reproduction, and behavior. Examples include altered feeding [35] with increased filtration rate [40] and morphological changes such as reduced carapace length and width [41]. Reproductive endpoints are particularly sensitive to SSRI exposure of *D. magna*. Several studies reported increased numbers of offspring following fluoxetine treatment [41,42]. At the molecular level, fluoxetine exposure altered antioxidant enzymes (superoxide dismutase, glutathione peroxidase) [40,41], often exhibiting a non-monotonic concentration–response effect. In contrast, lipid peroxidation, as indicated by elevated malondialdehyde levels, followed a concentration-dependent response [40]. Fluoxetine also affected acetylcholinesterase activity, implicating disruption of cholinergic neurotransmission [40]. SSRIs, by increasing serotonin levels, may also alter carbohydrate metabolism by upregulating hyperglycemic hormones [39,42]. Fluoxetine-exposed *D. magna* showed increased carbohydrate consumption via aerobic rather than anaerobic metabolism, enhancing energy efficiency but, at the same time, increasing sensitivity to hypoxia [42]. These metabolic alterations were supported by transcriptomic analyses [43]. Elevated respiration rates suggest increased maintenance costs, likely related to the activation of detoxification pathways [42]. Aerobic metabolism, however, leads to increased reactive oxygen species production (ROS), contributing to oxidative stress and potential protein damage, consistent with the reduced protein levels reported in some studies [41]. Furthermore, fluoxetine exposure has been linked to mitochondrial dysfunction, including membrane depolarization, electron leakage, and impaired redox homeostasis, all contributing to elevated ROS formation [41,44]. One of the metabolic pathways significantly affected by SSRIs and mianserin exposure in our study was cysteine and methionine metabolism, marked by increased levels of ketobutyrate and aminobutyrate. The latter may be changed into ophthalmic acid, an indicator of oxidative stress, reflecting glutathione depletion [45].

According to published studies, lipid metabolism, which is closely linked to growth, molting, and reproduction in *Daphnia* [46,47], is also disrupted by fluoxetine exposure. Reduced lipid droplet abundance was observed in post-spawning females, suggesting potential endocrine disruption [46]. Lipidomic analyses further demonstrated that environmentally relevant concentrations of fluoxetine (0.1 and 1 μg/L) increased glycerophospholipid levels (PC, PE, PS) while depleting other lipid classes. These phospholipids, primarily obtained by *Daphnia* from algal diets, are key sources of polyunsaturated fatty acids (PUFAs), including arachidonic acid, which are essential for *Daphnia*’s growth and reproduction [47]. Evidence from other studies further indicated that fluoxetine exposure not only upregulates serotonergic signaling but also modulates arachidonic acid pathways, leading to increased production of eicosanoids, lipid mediators derived from PUFA precursors [48]. These changes have been associated with altered offspring production, supporting the conclusion that SRRIs can disrupt lipid-based regulatory mechanisms in aquatic invertebrates. Our study also shows that exposure of *Daphnia* to 25 μg/L fluoxetine affects glycerophospholipid metabolism, increasing levels of PC and PE, consistent with previous studies.

Similar to fluoxetine, exposure of *D. magna* to sertraline (1 μg/L) affected the activity of antioxidant enzymes (superoxide dismutase and glutathione S-transferase) as well as acetylcholinesterase [49]. Environmentally relevant concentrations (0.1 and 10 μg/L) were also reported to promote growth and alter reproduction in *Daphnia*, increasing offspring number while reducing their size. Additionally, sertraline exposure delayed the first molt, which was accompanied by upregulation of *nvd1* and *hormone receptor* 3 genes; the former is involved in the initial step of *de novo* ecdysteroid synthesis from cholesterol, and the latter functions downstream in the ecdysteroid signaling pathway, associated with chitin turnover and molting behavior [37]. These findings are consistent with our results, which showed elevated levels of dehydrodesmosterol in all PhAC-treated groups compared to controls.

In our exposure studies, we also employed *S. ambiguum*, a widely distributed, ciliated protozoan commonly found in aquatic environments. As a primary consumer, it plays a vital role in the aquatic food web and can directly ingest contaminants from the surrounding water. This capacity makes it ecologically relevant and potentially useful in wastewater treatment processes, where it may contribute to contaminant removal [33]. *S. ambiguum* has been used as a model organism in ecotoxicological research for over 35 years. One of its major advantages is its relatively large cell size, which facilitates microscopic observations of sublethal effects, such as morphological alterations [50]. Furthermore, its short life cycle, ease of cultivation, and low maintenance cost make it an efficient and cost-effective tool for high-throughput toxicity screening [51].

Previous studies have demonstrated specific subcellular effects in *S. ambiguum* following exposure to PhACs. For instance, exposure to SSRI and mianserin resulted in pronounced vacuolization, likely due to the interaction of these PhACs with lysosomal membrane phospholipids [33]. This observation is consistent with the alterations in glycerophospholipid pathways observed in our study, which may have been related to increased membrane formation during vacuolization and intracellular trafficking.

There is growing evidence of the presence of serotonin receptors in protozoan species such as *Tetrahymena* and *Paramecium* [52]. While serotonin biosynthesis by these organisms has not been definitely confirmed [52], serotonin has been detected in several ciliates [53]. Functionally, serotonin has been shown to stimulate phagocytosis in *Paramecium* and *Tetrahymena* [53] and to promote ciliary regeneration in *Tetrahymena* [52]. Given that the expression of SERT in protozoa has not been confirmed, SSRIs are not expected to affect serotonin levels in *S. ambiguum* and are more likely to act through alternative molecular targets.

Importantly, antidepressants are increasingly recognized for their potential antiparasitic activity, as many exhibit cytotoxic effects against pathogenic protozoa. For examples, sertraline has been shown to induce mitochondrial membrane depolarization, a significant decrease in ATP levels, and, ultimately, irreversible damage and cell death in *Trypanosoma cruzi.* Similar effects were observed in *Leishmania donovani,* supporting the suggested mode of action. To the best of authors’ knowledge, there are no available data on the presence or expression of *SERT* in protozoa. One proposed molecular target of sertraline in those organisms is isocitrate dehydrogenase 2 (IDH2), an enzyme involved in the oxidative decarboxylation of isocitrate to 2-oxoglutarate, CO_2_, and NADPH, which are important for parasite resistance to oxidative stress [54]. Further evidence of SRRI-induced effects in protozoa comes from studies on *Paramecium caudatum*, where fluoxetine exposure (100–200 µg/L) led to decreased antioxidant enzyme activity (SOD and CAT) and increased lipid peroxidation, indicating oxidative stress as a key mechanism of its toxicity [55]. In our study, we observed disturbances in cysteine and methionine metabolism, marked by elevated levels of ketobutyrate and aminobutyrate. This metabolic shift suggested a response to oxidative stress. Cysteine is essential for the synthesis of glutathione, the primary intracellular antioxidant. Under conditions of oxidative stress or limited cysteine availability, cells may compensate by producing ophthalmic acid, an analogue of glutathione in which cysteine is replaced by aminobutyrate [56]. The increased concentration of aminobutyrate observed in our data may reflect this compensatory mechanism. Together with elevated ketobutyrate, a precursor of aminobutyrate, these changes were consistent with glutathione depletion and oxidative stress conditions. Thus, the metabolic profile we detected supports the notion that the studied system was under redox imbalance and relied on alternative pathways to maintain cellular homeostasis.

Additionally, mianserin, TeCA, has demonstrated antiparasitic properties by competitively inhibiting 3-hydroxy-3-methylglutaryl-coenzyme A reductase (HMGR), an enzyme critical for the synthesis of ergosterol, which is a major component of *Leischmania* cell membranes [57]. The alterations in steroid compounds observed in our study may be explained by impaired HMG-CoA reductase activity in relation to mianserin exposure, as suggested by the previously cited authors.

**Figure 5:**
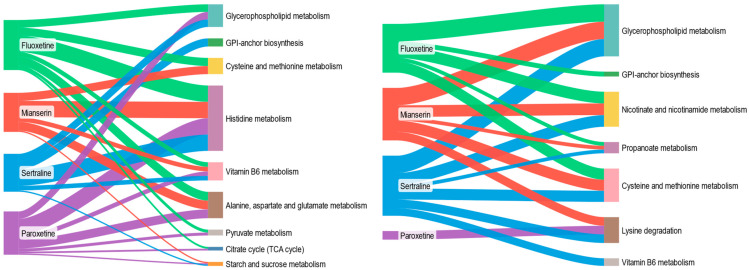
Metabolic pathways affected by each PhAC in *S. ambiguum* (**left**) and *D. magna* (**right**). The width of the lines in the Sankey diagrams corresponds to the magnitude of the impact on each metabolic pathway. Only pathways with FDR < 0.05 are presented.

Antidepressant exposure altered both overlapping and distinct metabolic pathways in *D. magna* and *S. ambiguum* (Figure 5). In both species, glycerophospholipid metabolism was consistently disrupted, indicating a shared sensitivity reflected by changes in membrane lipid composition or lipid signaling. Cysteine and methionine metabolism was also affected in both organisms, suggesting conserved responses in sulfur amino acid metabolism related to oxidative stress. However, *D. magna* showed unique alterations in lysine degradation and nicotinate and nicotinamide metabolism, pointing to changes in protein turnover and NAD^+^-related redox activity that were not observed in *S. ambiguum*. Conversely, *S. ambiguum* displayed consistent alterations in alanine, aspartate, and glutamate metabolism and more frequent enrichment of histidine metabolism, suggesting disruptions in protein synthesis and oxidative defense, as well as in the histaminergic system and in energy homeostasis. These findings indicate both conserved and divergent metabolic responses to antidepressants, shaped by the biological complexity and metabolic architecture of each organism.

Interestingly, PhACs within the same class, i.e., SSRIs, were found to differentially affect the levels of certain metabolites, while in other cases, SSRIs produced effects similar to those of minserin, a TeCA. These observations align with previous studies reporting divergent metabolic responses to PhACs with similar primary mechanisms of action. For example, sertraline and fluoxetine showed opposing changes in 5-HIAA (a major degradation product of serotonin) levels, i.e., an increase and decrease, respectively [58]. It was suggested that such variability may reflect differences in PhAC bioaccumulation and may also be related to their secondary modes of action.

An untargeted metabolomics approach enabled the comprehensive identification of multiple altered metabolic pathways. However, it remains insufficient to clearly differentiate transient metabolic alterations from long-lasting, potentially adaptive changes. Understanding this distinction is crucial for selecting robust biomarkers that reliably indicate ecotoxicological risks. Based on the available literature, many observed metabolomic alterations, particularly in *Spirostomum*, remain difficult to fully explain. Nonetheless, these findings provide a valuable foundation for formulating hypotheses regarding potential molecular targets and mechanisms of toxicity.

## 4. Materials and Methods

### 4.1. Culturing and Exposure Conditions for Test Organisms

*S. ambiguum* cultures were maintained in inorganic Tyrode solution adjusted to pH 7.4. Cultivation was carried out in 250 mL glass beakers containing 200 mL of medium, each inoculated with 1000 protozoan cells. Incubation was conducted at 25 °C in darkness. *S. ambiguum* was exposed to fluoxetine, sertraline, paroxetine, or mianserin at a concentration of 100 µg/L for 48 h. This concentration, which is higher than environmentally relevant levels, was chosen to allow for the detection of metabolomic alterations within a short exposure period. Importantly, it remains well below reported EC_50_ values, thereby ensuring that observed effects were not the result of overt toxicity [33]. *D. magna* were obtained from a culture established with the Daphtoxkit F magna [59]. The test organisms were 5 days old. They were cultured under laboratory conditions at 25 °C in complete darkness. Groups of 20 individuals were exposed to fluoxetine, sertraline, paroxetine, or mianserin at a concentration of 25 µg/L for 48 h in 150 mL glass vessels containing 100 mL of test solution. Throughout the 48-h testing period, they were not provided with food. This duration represents the maximum time *D. magna* can remain viable without feeding. The mortality in all groups was similar and less than 10%.

### 4.2. Sample Preparation

Sample preparation for LC-MS/MS analysis was conducted separately for *S. ambiguum* and *D. magna,* using a procedure similar to that described previously [33]. For *S. ambiguum*, 100 µL of culture containing approximately 500 cells was transferred to Eppendorf tubes and mixed with 200 µL of acetonitrile. Samples were vortexed for 10 min, frozen at −20 °C for 10 min, and centrifuged at 10,000× *g* for 5 min. The resulting supernatant (150 µL) was diluted with 375 µL of mobile phase and transferred to vials for analysis.

For *D. magna*, 10 dried individuals were transferred to Eppendorf tubes, frozen at −20 °C, and homogenized with 50 µL of deionized water and 150 µL of acetonitrile using a manual homogenizer. Samples were centrifuged at 10,000× *g* for 1 min, then frozen again. Finally, 250 µL of supernatant was diluted with 375 µL of mobile phase and transferred to vials for analysis.

### 4.3. Instrumental Analysis

Instrumental metabolomic analysis was carried out using an Ultimate 3000 high-performance liquid chromatography (HPLC) system (Thermo Fisher Scientific, Waltham, MA, USA) with an autosampler, column thermostat, and mixer, coupled to a high-resolution Orbitrap Focus mass spectrometer (Thermo Fisher Scientific, Waltham, MA, USA), as described previously [20]. Chromatographic separation was performed on two columns: SeQuant Zic HILIC (100 mm × 2.1 mm, 5 μm) (Merck, Darmstadt, Darmstadt, Germany) and Kinetex PFP (100 mm × 4.6 mm, 5 μm) (Phenomenex, Torrance, CA, USA). The mobile phases consisted of (A) HPLC-grade water with 0.1% formic acid and (B) acetonitrile with 0.1% formic acid. The column temperatures were maintained at 40 °C and the flow rate was 0.3 mL/min. The injection volumes were 10 µL. The gradient on Kinetex PFP was as follows: 20% B at 0 min, 20% B at 2 min, 90% B at 20 min, 90% B at 30 min, 20% B at 31 min, and 20% B at 35 min. The gradient on the HILIC column was 90% B at 0 min, 90% B at 2.5 min, 50% B at 18 min, 50% B at 28 min, and 90% B at 34 min. Electrospray ionization (ESI) was performed in both positive and negative modes. Mass spectra were recorded in a *m*/*z* range of 75–1100 with a resolution of 70,000. Fragmentation spectra were acquired using collision energy of 20, 40 and 60 V (resolution 17,500).

### 4.4. Statistical Analysis

The metabolomic data were analyzed with a Compound Discoverer 3.2 (Thermo Fisher Scientific, Waltham, MA, USA) (peak detection and annotations of metabolites) and MetaboAnalyst 6.0 (statistical analyses and data visualization). The data were log-transformed (base 10). Principal component analysis (PCA) was applied to evaluate the quality of the obtained data by evaluating clustering in the quality control samples. Furthermore, PCA, as an unsupervised method, was also used in data exploration. A heatmap obtained with row-standardized features was used to visualize patterns in changes of metabolite levels across samples. Euclidean distance was used as a measure of similarity, whereas Ward’s method was used for clustering. Metabolic Pathway Analysis (with the *D. pulex* library for *D. magna* and the *Paramecium tetraurelia* library for *S. ambiguum*) in MetaboAnalyst 6.0 was used to elucidate possible biological processes affected by exposure to antidepressants. FDR correction was applied to account for multiple testing and control the false discovery rate.

## 5. Conclusions

Untargeted metabolomics based on LC-MS effectively captured sublethal biochemical responses to antidepressants in two early trophic-level aquatic organisms. Both *D. magna* and *S. ambiguum* exhibited alterations in key metabolic pathways following exposure to fluoxetine, paroxetine, sertraline, and mianserin. Shared disruptions, such as those in glycerophospholipid metabolism and cysteine and methionine metabolism, suggested some conserved responses to xenobiotic exposure. However, the more distinct pathway changes observed in *D. magna* compared to *S. ambiguum* pointed to differences in metabolic capacity, biological complexity, or PhAC processing between taxa. These findings emphasize the value of incorporating multiple organism types into ecotoxicological studies and demonstrate the sensitivity of metabolomic approaches in assessing the ecological risks of antidepressants. Among the four antidepressants tested, sertraline in *D. magna* and fluoxetine in *S. ambiguum* exerted the most extensive metabolomic perturbations, as evidenced by the highest number and pathway impact scores. In *D. magna*, fluoxetine and mianserin produced similar metabolic profiles, largely overlapping with those of sertraline, whereas paroxetine affected only a single pathway, indicating minimal impact. In *S. ambiguum*, paroxetine and mianserin elicited comparable responses, also overlapping with those of fluoxetine, while sertraline triggered the fewest changes. These findings suggest both compound-specific effects and a conserved metabolic response pattern among the antidepressants used. Metabolomics appears to be a highly promising tool for assessing the environmental toxicity of pharmaceuticals; however, further research is needed—particularly studies that can identify which specific pathway disruptions pose significant risks to organismal health.

This study has several limitations. First, untargeted metabolomics did not allow for clear differentiation between transient or adaptive metabolic responses and those that were potentially deleterious. Thus, it could not predict which metabolic alterations may lead to long-term physiological effects. To address this limitation, future research should incorporate long-term exposure studies, including transgenerational monitoring and phenotypic anchoring—such as reproductive performance, behavioral assays, and developmental endpoints. The integration of multi-omics approaches (e.g., transcriptomics, proteomics) may further improve biological interpretation and help identify consistent markers of adverse outcomes. Second, due to the lack of species-specific databases, pathway analysis was performed using the *Paramecium tetraurelia* and *Daphnia pulex* libraries. These species were chosen for their phylogenetic proximity to the studied organisms. Preliminary comparisons with other available libraries showed comparable affected pathways. While major biases are unlikely, the possibility of some pathway-level misinterpretation cannot be fully excluded. Another limitation to consider was the use of antidepressant concentrations (100 µg/L for *S. ambiguum* and 25 µg/L for *D. magna*) which exceeded typical environmental levels, i.e., usually in the nanogram per liter range. Nonetheless, concentrations reaching several micrograms per liter have been reported in wastewater treatment plant influents (e.g., up to 39.7 µg/L for paroxetine) [60]. Given the increasing global consumption of antidepressants and the limited removal efficiency of conventional WWTP, such elevated concentrations may become increasingly prevalent in the aquatic environment. Therefore, while the concentrations used in this study may not reflect current average environmental levels, they represent a plausible worst-case exposure scenario. Additionally, these concentrations were selected to ensure the detection of metabolic alterations within the short exposure durations used. This approach was consistent with previous metabolomic studies demonstrating that the subtle biological effects observed at environmentally relevant concentrations often become statistically significant only at higher levels [20,61]. As such, our data provide mechanistic insights into early molecular responses, supporting hypothesis generation and guiding further research using environmentally realistic exposures.

Metabolomics, while not yet routinely integrated into regulatory toxicology or environmental risk assessment frameworks, is increasingly being recognized as a powerful and complementary approach for toxicological evaluations. Compared to conventional toxicity tests, metabolomics can offer faster and more cost-effective assessments and often reduces the number of organisms required, aligning with the principles of the 3Rs (Replacement, Reduction, and Refinement). Importantly, by capturing early biochemical disruptions, this approach enables the identification of both species-specific and shared mechanisms of toxicity, improving cross-species extrapolation and informing ecological risk predictions. Nonetheless, the effective integration of metabolomics into environmental toxicology requires rigorous standardization—both in experimental design (e.g., exposure concentrations, duration) and in data processing and reporting—to ensure reproducibility, comparability, and regulatory relevance [62,63].

## Data Availability

Data are available on request.

## Supplementary Materials

The following supporting information can be downloaded at: https://www.mdpi.com/article/10.3390/molecules30142952/s1, Figure S1. Heatmaps of all significantly altered metabolites, selected based on ANOVA, in *S. ambiguum* following fluoxetine (F), mianserin (M), paroxetine (P) and sertraline (S) exposure. K-control group. Red indicates higher abundance compared to other exposure conditions, while green represents lower relative abundance. Figure S2. Heatmaps of all significantly altered metabolites, selected based on ANOVA, in *D. magna* following fluoxetine (F), mianserin (M), paroxetine (P) and sertraline (S) exposure. K-control group. Red indicates higher abundance compared to other exposure conditions, while green represents lower relative abundance. Raw data were also presented as xlsx file.
