# Supplementary material for: Environmental Antidepressants Disrupt Metabolic Pathways in Spirostomum ambiguum and Daphnia magna: Insights from LC-MS-Based Metabolomics
Source: Molecules. 2025 Jul 13;30(14):2952. doi: 10.3390/molecules30142952 (PMC12300871; doi:10.3390/molecules30142952)
Supplement: Supplementary file 1 [file molecules-30-02952-s001.zip › molecules-3730591-supplementary materials.pdf]

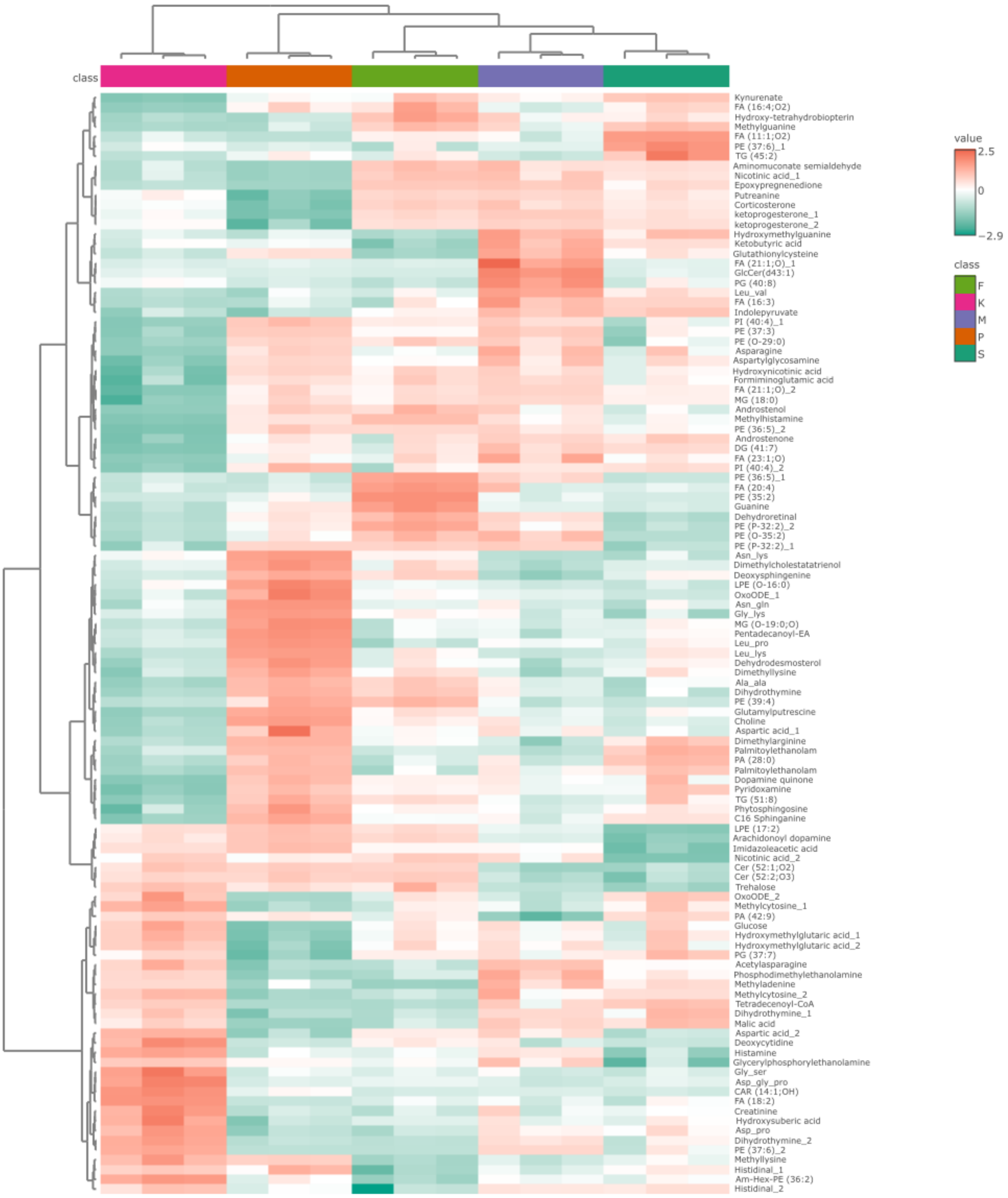

Figure S1. Heatmaps of all significantly altered metabolites, selected based on ANOVA, in *S. ambiguum* following fluoxetine (F), mianserin (M), paroxetine (P) and sertraline (S) exposure. K-control group. Red indicates higher abundance compared to other exposure conditions, while green represents lower relative abundance.

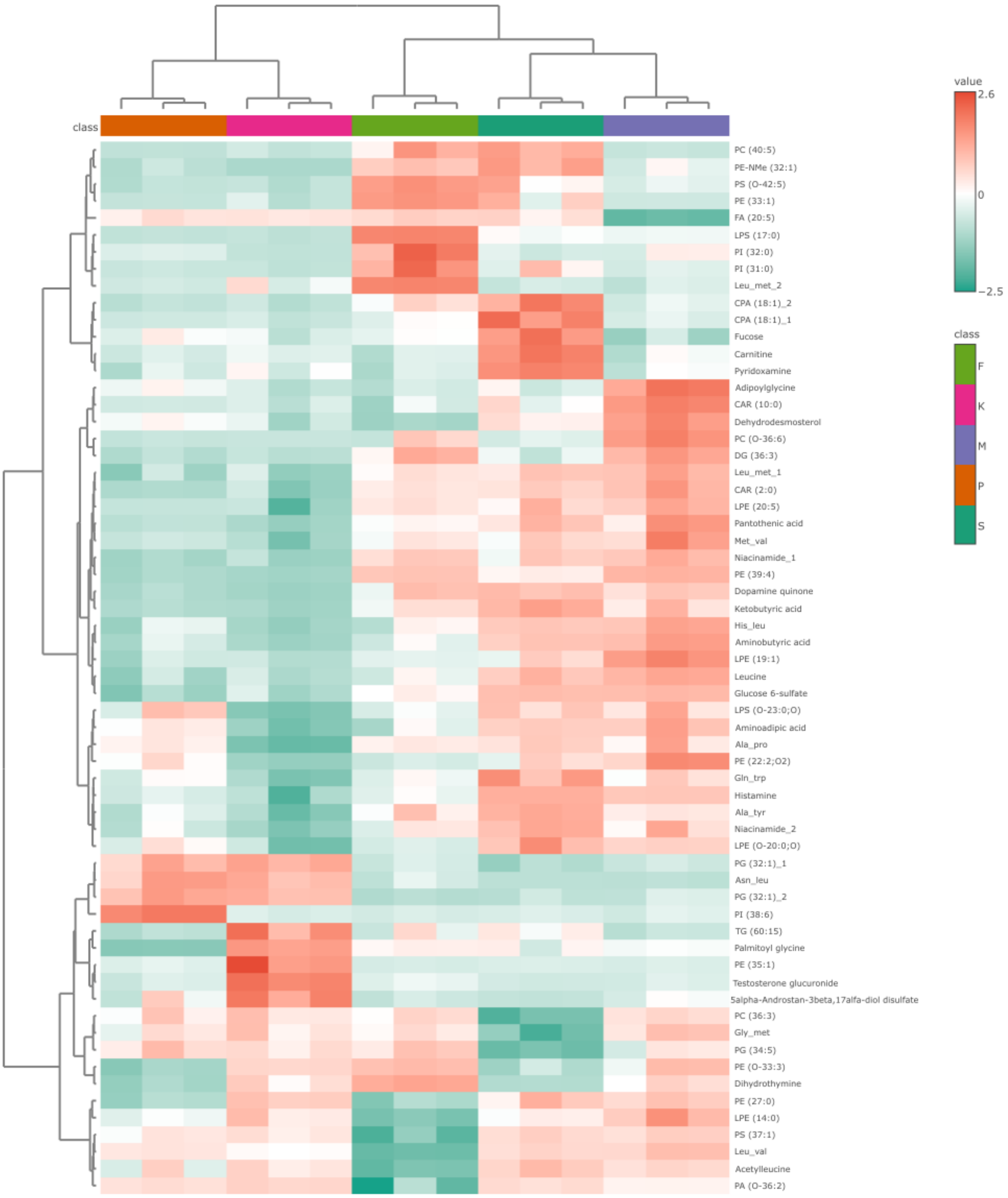

Figure S2. Heatmaps of all significantly altered metabolites, selected based on ANOVA, in *D. magna* following fluoxetine (F), mianserin (M), paroxetine (P) and sertraline (S) exposure. K-control group. Red indicates higher abundance compared to other exposure conditions, while green represents lower relative abundance.
